# Autonomous assessment of spontaneous retinal venous pulsations in fundus videos using a deep learning framework

**DOI:** 10.1038/s41598-023-41110-8

**Published:** 2023-09-02

**Authors:** Amirhossein Panahi, Alireza Rezaee, Farshid Hajati, Sahar Shariflou, Ashish Agar, S. Mojtaba Golzan

**Affiliations:** 1 Faculty of New Sciences and Technologies, University of Tehran, Tehran, Iran; 2 Intelligent Technology Innovation Lab (ITIL) Group, Institute for Sustainable Industries and Liveable Cities, Victoria University, Footscray, Australia; 3 Vision Science Group, Graduate School of Health, University of Technology Sydney, Ultimo, Australia; 4 Ophthalmology Department, Prince of Wales Hospital, Sydney, NSW Australia; 5 Department of Ophthalmology, University of New South Wales, Sydney, NSW Australia; 6 Marsden Eye Specialists, Sydney, NSW Australia

**Keywords:** Diseases, Eye diseases

## Abstract

The presence or absence of spontaneous retinal venous pulsations (SVP) provides clinically significant insight into the hemodynamic status of the optic nerve head. Reduced SVP amplitudes have been linked to increased intracranial pressure and glaucoma progression. Currently, monitoring for the presence or absence of SVPs is performed subjectively and is highly dependent on trained clinicians. In this study, we developed a novel end-to-end deep model, called U3D-Net, to objectively classify SVPs as present or absent based on retinal fundus videos. The U3D-Net architecture consists of two distinct modules: an optic disc localizer and a classifier. First, a fast attention recurrent residual U-Net model is applied as the optic disc localizer. Then, the localized optic discs are passed on to a deep convolutional network for SVP classification. We trained and tested various time-series classifiers including 3D Inception, 3D Dense-ResNet, 3D ResNet, Long-term Recurrent Convolutional Network, and ConvLSTM. The optic disc localizer achieved a dice score of 95% for locating the optic disc in 30 milliseconds. Amongst the different tested models, the 3D Inception model achieved an accuracy, sensitivity, and F1-Score of 84 ± 5%, 90 ± 8%, and 81 ± 6% respectively, outperforming the other tested models in classifying SVPs. To the best of our knowledge, this research is the first study that utilizes a deep neural network for an autonomous and objective classification of SVPs using retinal fundus videos.

## Introduction

Spontaneous retinal venous pulsations (SVP) are rhythmic changes of the central retinal vein that are visible on (or adjacent) to the optic disc^1^. Their frequency is also closely matched to the cardiac frequency^2^. SVPs originated as a result of a complicated interplay between systemic blood pressure, intraocular pressure, cerebrospinal fluid pressure (also known as intracranial pressure (ICP)), vessel structure, stiffness, and diameter^3^. Due to this interaction with the intraocular and intracranial space, SVPs hold clinically significant information relevant to a range of eye and brain diseases such as glaucoma^4^, intracranial hypertension^5^, and visual impairment intracranial pressure (VIIP)^4–6^. Despite the clinical significance of SVP monitoring, an autonomous and objective method for SVP assessment is currently not available, and SVPs are monitored subjectively by an expert clinician.

In the eye, SVP assessment has been recognized as an important marker for glaucoma onset and progression^7^. Previous studies have shown that SVPs are less evident in glaucoma and glaucoma suspects than in healthy individuals^8,9^. Morgan et al.^8^ show that SVP was visible in 54% of glaucoma cases, 75% of glaucoma suspects, and 98% of healthy instances. Legler and Jonas showed SVP presence in 64.1% of glaucoma subjects and 75.3% of healthy cases^9^. Several factors have been suggested as to why SVPs are reduced in glaucoma and glaucoma suspects. Those include an alteration in the ocular perfusion pressure^10^, fluctuations in translaminar pressure gradient^11^, and downstream increased vascular resistance^12^.

In the brain, the cerebrospinal fluid (CSF) comes into contact with the central retinal vein via the optic nerve sheath^13^. As a result, fluctuations in the CSF, directly transverse to the central retinal vein, leading to changes in the SVP amplitude. An increase in CSF pressure (aka, ICP) is a characteristic of several neurological conditions such as trauma^14^, intracranial mass lesions^15^, idiopathic intracranial hypertension^16^, hydrocephalus^17^, stroke^18^, and VIIP experienced astronauts^19^. Previous studies have shown that SVPs are reduced with increasing ICP and may be re-established by raising the IOP to above the ICP by about 5–10 mm Hg^20^. Collectively, the presence or absence of SVPs can be a clinical indicator for normal or abnormal levels of ICP, respectively.

Both statistical and structural image analysis models have been proposed to quantify SVPs. McHugh et al.^21^ used the Spectralis optical coherence tomography device to record an infrared video, of 10 s in length, from each retina, centered on the optic disc. The presence of SVPs was assessed subjectively using the grading system suggested by Hedges et al.^22^. Principal Component Analysis (PCA) was applied by Moret et al.^3^ to sequential retinal images to detect SVP. They found that the most vital pulsatile signs were hidden in the first 5 to 10 components. Shariflou et al.^23^ applied a custom-written algorithm based on contrast-limited adaptive histogram equalization (CLAHE) and a method proposed by Fischer et al.^24^ to measure SVP amplitudes. While their approach enabled an objective measurement of SVPs, it was heavily resource-intensive. Despite previous attempts, to the best of our knowledge, an autonomous method to detect SVPs using fundus videos does not exist. In this study, we have developed a deep neural network trained on retinal fundus videos to autonomously detect the presence or absence of SVPs.

## Materials and methods

### Dataset

The retinal images used in the study were obtained from publicly available repositories. For retinal videos, the study was performed in accordance with the guidelines of the tenets of the Declaration of Helsinki and approved by the University of Technology Sydney’s Human Research Ethics Committee (ETH17-1392). Informed consent was obtained from each participant following an explanation of the nature of the study.

Two distinct sets of fundus videos and images were used to develop and test the performance of our proposed model. For fundus videos, a total of 185 were collected from 113 participants attending the Marsden Eye Clinic. All participants were recruited subject to the following inclusion/exclusion criteria:

- Inclusion criteria
  1. Adults (i.e., over 18 years of age)
  2. A normal fundus on ophthalmoscopy with no visible vascular changes.
  3. Clear ocular media with visual acuity better than 6/12.
- Exclusion criteria
  1. Persistent vision loss, blurred vision, or floaters.
  2. History of laser treatment of the retina or injections into either eye, or any history of retinal surgery.
  3. Anomalies of the ocular media that would preclude accurate imaging.
  4. Participant is contraindicated for imaging by fundus imaging systems used in the study (e.g. hypersensitive to light or on medication that causes photosensitivity)

**Figure 1:**
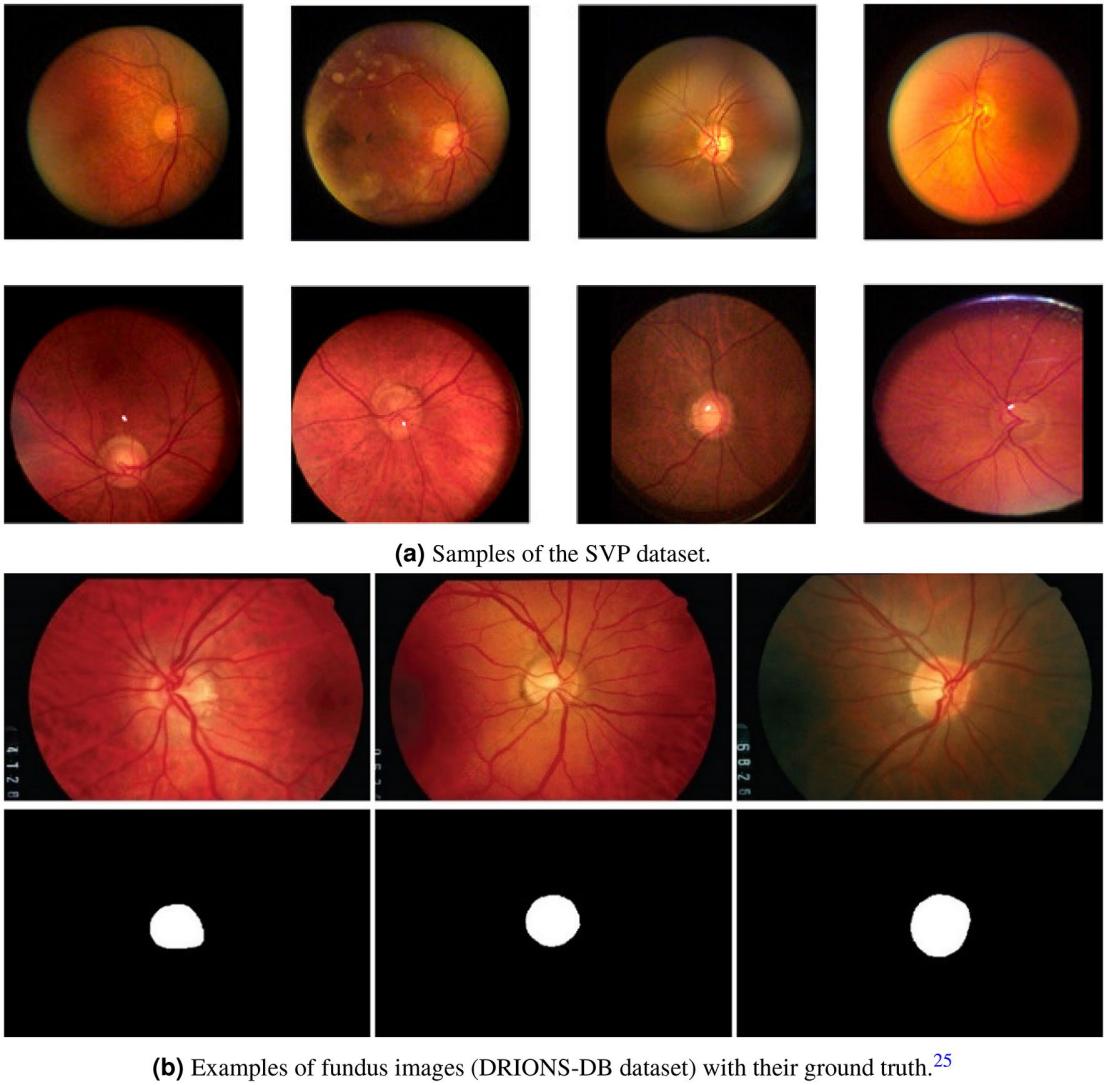
Some samples from SVP and DRIONS-DB datasets.

Participants had a dilated fundoscopy and a minimum 3-second recording (30 frames per second at a 46/60 degrees’ field of view of the retina and 2.2 image magnification) centered on the optic disc Fig. 1a. Co-authors SMG and SS reviewed all videos and marked SVPs as present or absent. Occurrence of SVPs were only assessed within one-disc diameter of the optic nerve head. Co-author AA adjudicated any disagreement in the assessment between the two graders.

For fundus images, we used the DRIONS-DB database^25^, a public dataset containing 110 fundus images with their annotated ground truth, for training the optic disc localization model (Fig. 1b).

### SVP classification

**Figure 2:**
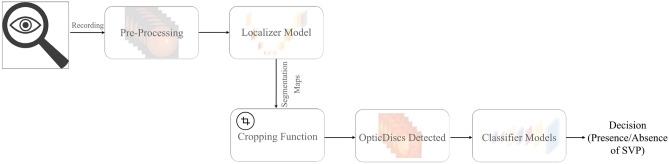
The overall structure of the U3D-Net.

To classify SVPs, we developed an end-to-end deep model called U3D-Net. Figure 2 shows the overall structure of the model. The U3D-Net receives fundus videos as input and classifies SVPs as present or absent. The U3D-Net consists of two main blocks: Optic Disc Localizer and Classifier. Since SVP occurs on (or adjacent to) the optic disc, the U3D-Net has been tuned to focus on the optic disc. For this purpose, the U3D-Net has an accurate and fast localizer that processes individual video frames and locates the optic disc in each image. The order of the frames, due to their synchronization with the cardiac frequency, is also an essential factor. This has been taken into account in the design of the localizer, which feeds sequential frames into the classifier. Therefore, SVPs are classified based on a batch of 30 sequential frames.

### Optic disc localizer

SVPs are mainly observable on the central retinal vein located on (or adjacent to) the optic disc. Therefore, prior to analyzing retinal videos for the presence or absence of SVPs, we developed a model that could localize optic discs in an image. For the purpose of our study, attention mechanisms^26^ with recurrent residual convolutional layers, which are depth-wise separable^27^, were used. A depth-wise separable layer decreases the computational cost in the network. The process includes a depth-wise and a spatial convolution operated separately across every input data channel. Following this, it is supported by a pointwise $1\times 1$ kernel convolution. To obtain the outcome of each channel $(O_1, O_2, O_3, O_4)$, each of the convolution kernels $(K_1, K_2, K_3, K_4)$ is convolved with one of the input channels $(I_1, I_2, I_3, I_4)$. Ultimately, the outcomes from different kernels are fused into one. The output of the $i-th$ kernel, $O_i$, is defined as

1 $$\begin{aligned} O_i = K_i \otimes L_i \end{aligned}$$

where $K_i$ and $O_i$ are convolution kernels and the outcome of each channel convolution kernels and outcome of each channel, respectively.

**Figure 3:**
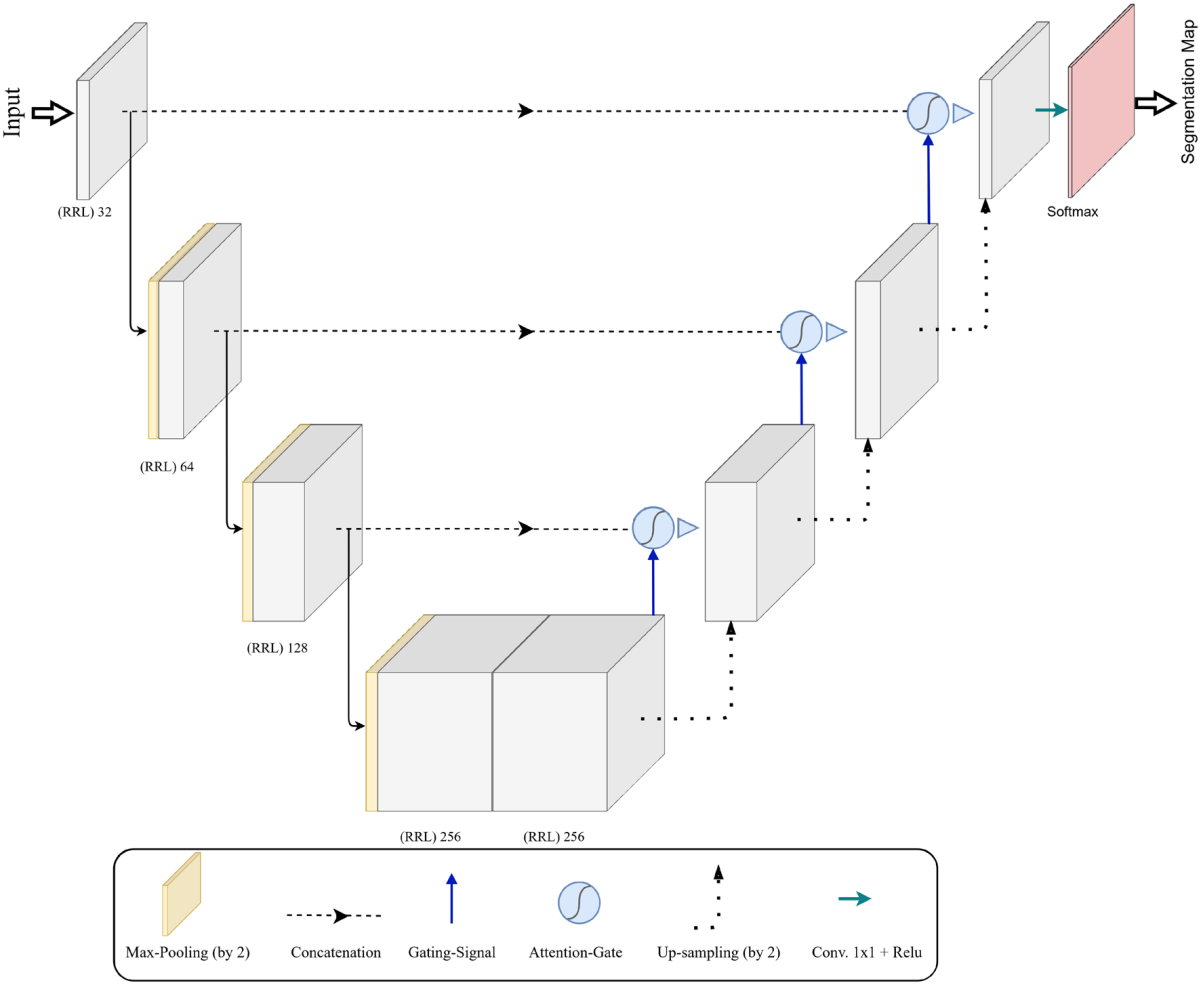
Proposed optic disc localizer. The number of filters of Depth-wise 2D Convolutional layers has been shown.

Equation (1) establishes the number of convolution operations needed for depth-wise separable layers. Our proposed architecture for optic disc localizer (Fig. 3) contains recurrent residual layers and an attention mechanism. In this architecture, we have eliminated and modified the original U-Net^28^ copying and cropping block and have employed a concatenation operation, resulting in a highly developed structure and improved proficiency. The fundamental idea of recurrent connections is to reuse maps or weights and keep some data. The output of a depth-wise separable convolution layer returns to the layer’s input before passing it to the next layer. Also, a residual unit assists in avoiding vanishing gradient problems during the training. Hence, feature extraction with recurrent residual convolutional layers ensures a more compelling feature representation, enabling us to design a more accurate optic disc localizer. The localizer model trained with Attention Gates (AGs)^26^ thoroughly learns to ignore unnecessary areas in an input image and focus on distinctive features valuable for optic disc detection. AGs can be mixed with recurrent residual convolutional layers with minimum computational cost while improving the model’s accuracy.

**Figure 4:**
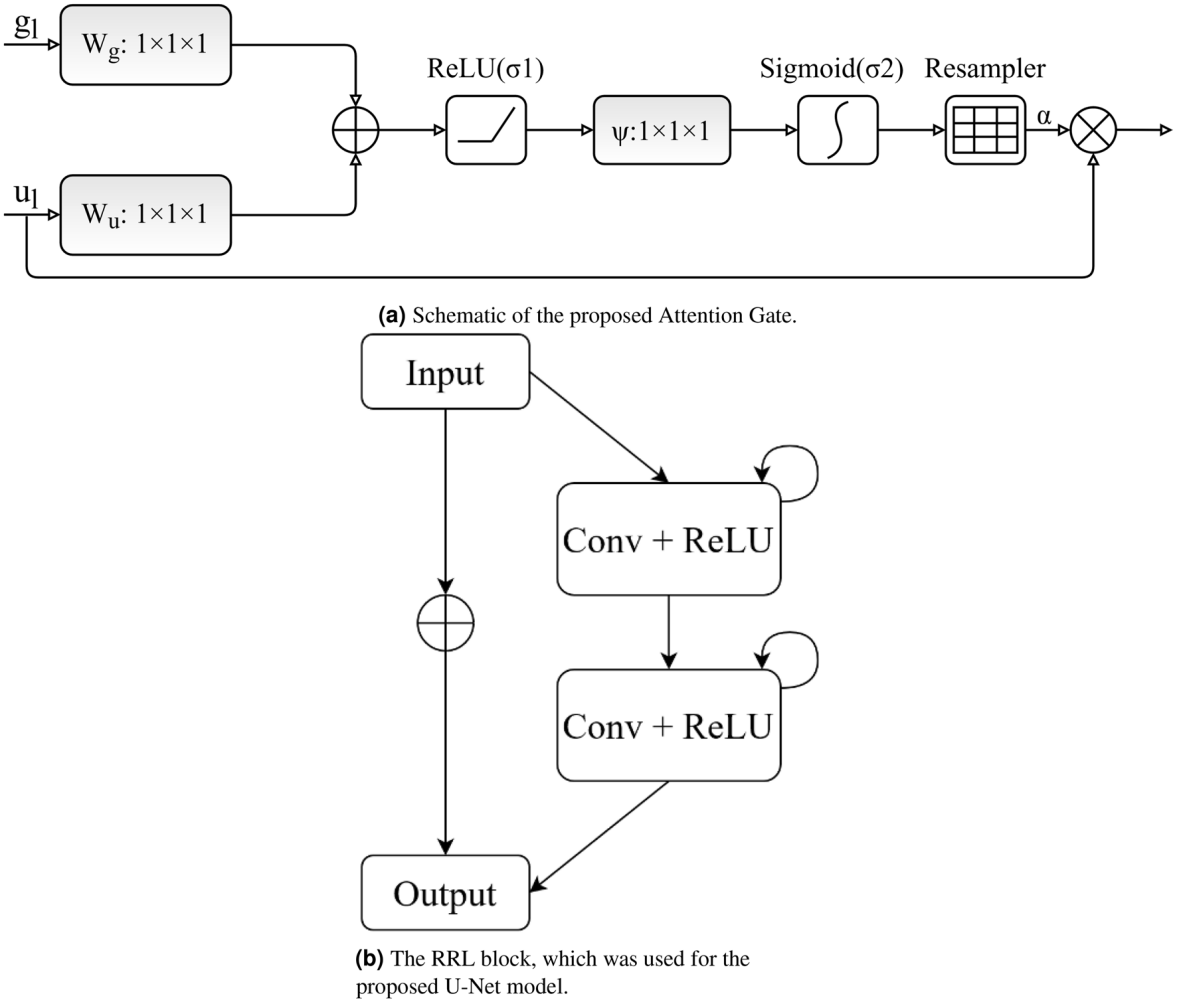
The details of RRL block and attention gate that are used in optic disc localizer model.

Figure 4a displays the proposed AG. Attention values are computed for each pixel (u). We assumed that $u_{l}^{down}$ and $u_{l}^{up}$ are represented as $u_l$ and $g_l$, respectively. The gating signal $g_l$ specifies the attention region per pixel. The additive attention^29^ is utilized to acquire the attention coefficient to achieve higher accuracy. The additive formula presents as follows:

2 $$\begin{aligned} Q_L = \psi (\sigma _1(W_u u_l + W_g g_l + b_g) + b)\psi , \alpha _1 = \alpha _2 (Q_L) \end{aligned}$$

where *Wg* is the weight $\sigma _1$ and $\sigma _2$ represent the activation functions of ReLU and sigmoid, respectively, and *bg* and $b_\psi$ denote the bias. The *AG* parameter is updated and trained based on the backpropagation technique rather than utilizing the sampling-based update process^30^. Finally, the result of *AGs* is the multiplication of the attention coefficient and the feature map are shown as follows:

3 $$\begin{aligned} c_l^{up} = \alpha \times u_1 \end{aligned}$$

The construction of the *RRL* block is illustrated in Fig. 4b. Localization of the optic disc encompasses contracting and expansive paths. The input of the localization block, which is individual video frames, is initially passed through a depth-wise separable convolutional layer with $3 \times 3$ filters. Then, the recurrent convolutional layers are utilized, and the final output of each recurrent convolution layer is passed on to the residual layer. We applied a time step of 1 second, indicating one forward convolution layer supported by one recurrent convolutional layer. Next, the ReLU activation function and max-pooling operation are applied, reducing the input width and height. The image resolution is reduced by passing the image through the sequence of layers multiple times. The same convolution layers and settings are used on the expansive side, with up-sampling layers, which lead to increased image resolution. Information obtained from the contracting path is utilized in the attention gate to remove noisy and unnecessary responses in skip connections. This is implemented directly before the concatenation process to merge just relevant and important activations.

**Figure 5:**
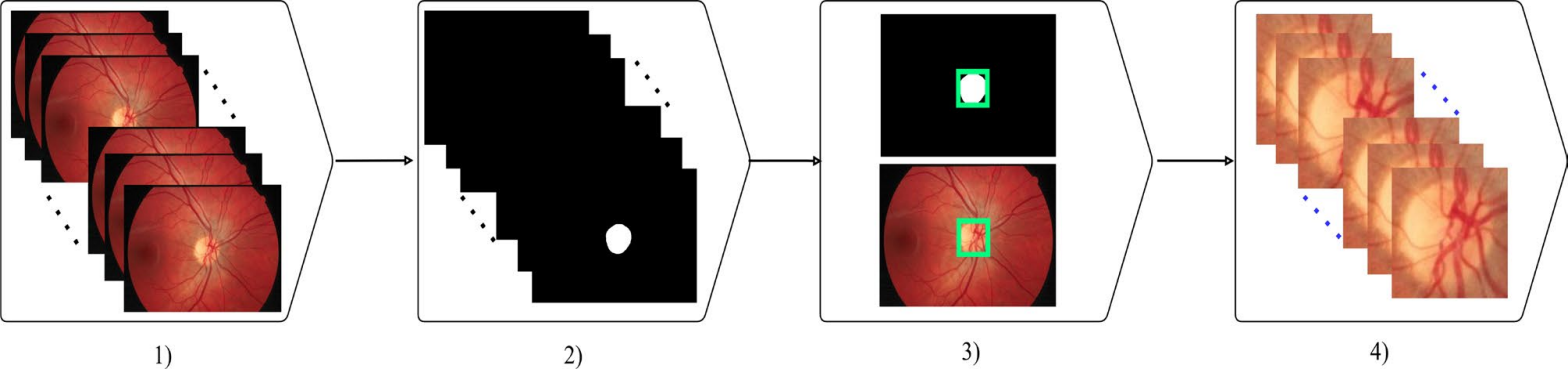
Optic disc localization steps: video frame extraction, creating segmentation maps by U-Net, localizing the optic discs using the segmentation maps, and building cropped optic disc videos.

The optic disc localizer model’s input is video frames, and the output is the segmentation map of the optic discs. As shown in Fig. 5, by calculating the coordinates area of the optic discs (white pixels) from the segmentation map, the region of the optic discs has been characterized. Finally, by applying a function to the frames of the video, the optic disc region will be cropped as a sequence form.

75% part of the DRIONS-DB dataset was used for training, 20% for validation, and the remaining 5% for testing the localizer model. An initial learning rate of 0.003 was used with a batch size of 6 and 100 epochs of training. In order to update the weights of the network iteratively, the RMSprop algorithm was used. In order to train and evaluate the optic disc localization model, the Dice loss function was selected since it is commonly used in medical image segmentation. By learning an effective feature representation and weight parameter, the model learned how to locate optic discs in fundus images accurately.

### Classifier

Following localization of the optic disc, sequential frames of the input video are then passed on to the classifier block of the U3D-Net. Each video frame was resized to 64$\times$64 pixels and converted into grayscale to decrease computational time and complexity. Different deep learning networks in this paper have been evaluated: 3D Inception, 3D Dense-ResNet, 3D ResNet, Long-term Recurrent Convolutional Network (LRCN), and ConvLSTM. These networks were chosen as they have been used widely for medical image and video application tasks. All networks comprise some layers such as convolutional layers, pooling layers, and a fully connected layer that makes a label for the input data. Also, the final performance of each network has been analyzed in the next part. Two characteristics are essential for video classification: spatial (static) features within each frame and temporal (dynamic) features between sequential frames. To evaluate the performance of each classifier model in classifying SVPs, and to increase the number and variety of fundus videos, data augmentation using a 180 degrees rotation of original videos was used. Also, to remove any bias due to the chosen sets, a K-fold cross-validation was used in which the SVP dataset is indiscriminately divided into five equal-sized folds (partitions). In this procedure, a single fold is chosen to serve as the test set, while the rest of the four folds are combined to form the training set. This process is repeated five times, with each fold serving as a test set once. Rotating the test set between the folds guarantees that the algorithm is assessed on various subsets of the data. Finally, the average of the results is calculated. This enables a more reliable estimation of its performance and generalization capabilities. The structure of each classifier model based on different deep learning structures with their detail has been presented in what follows.

#### 3D inception

**Figure 6:**
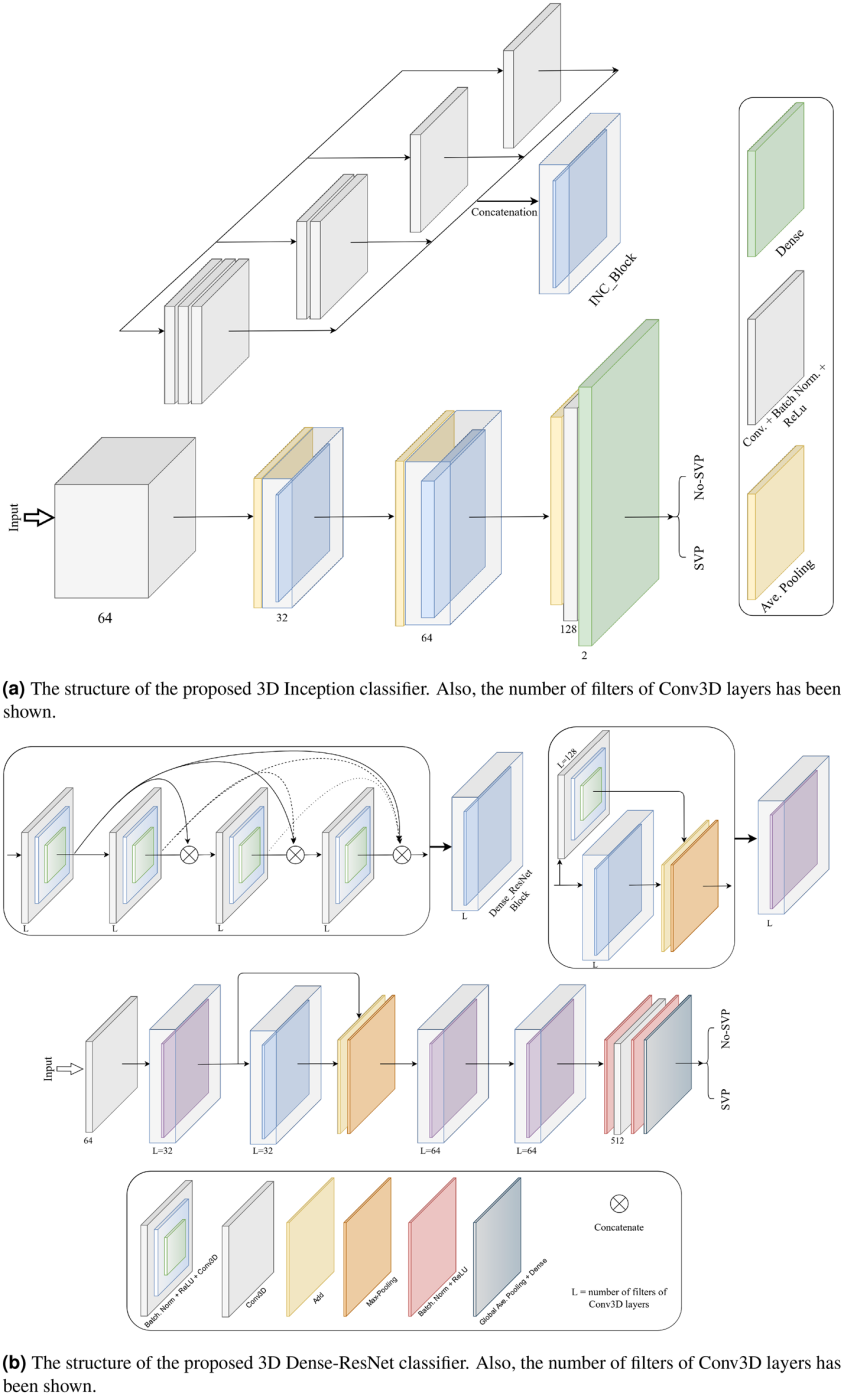

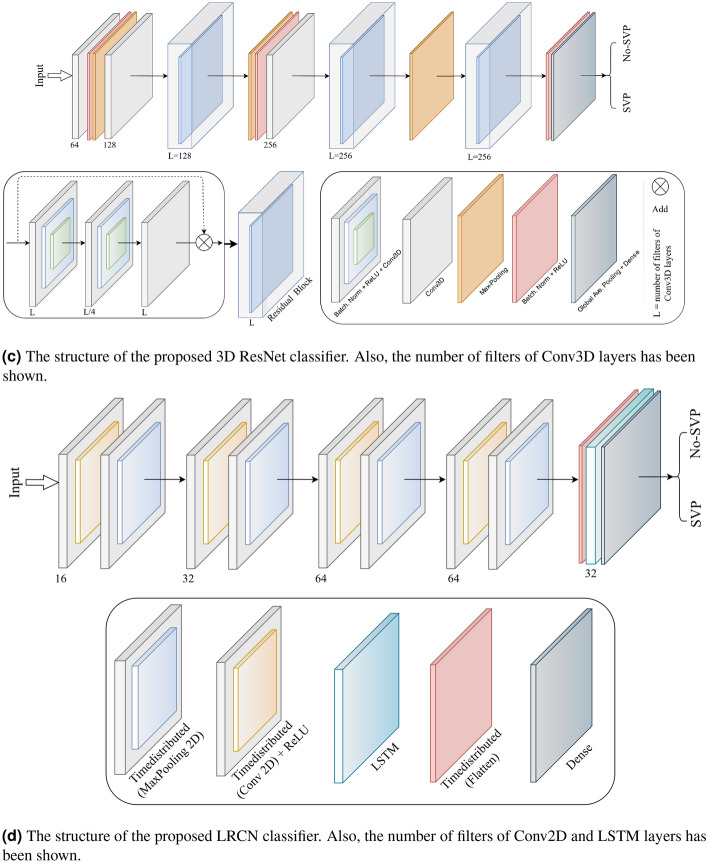

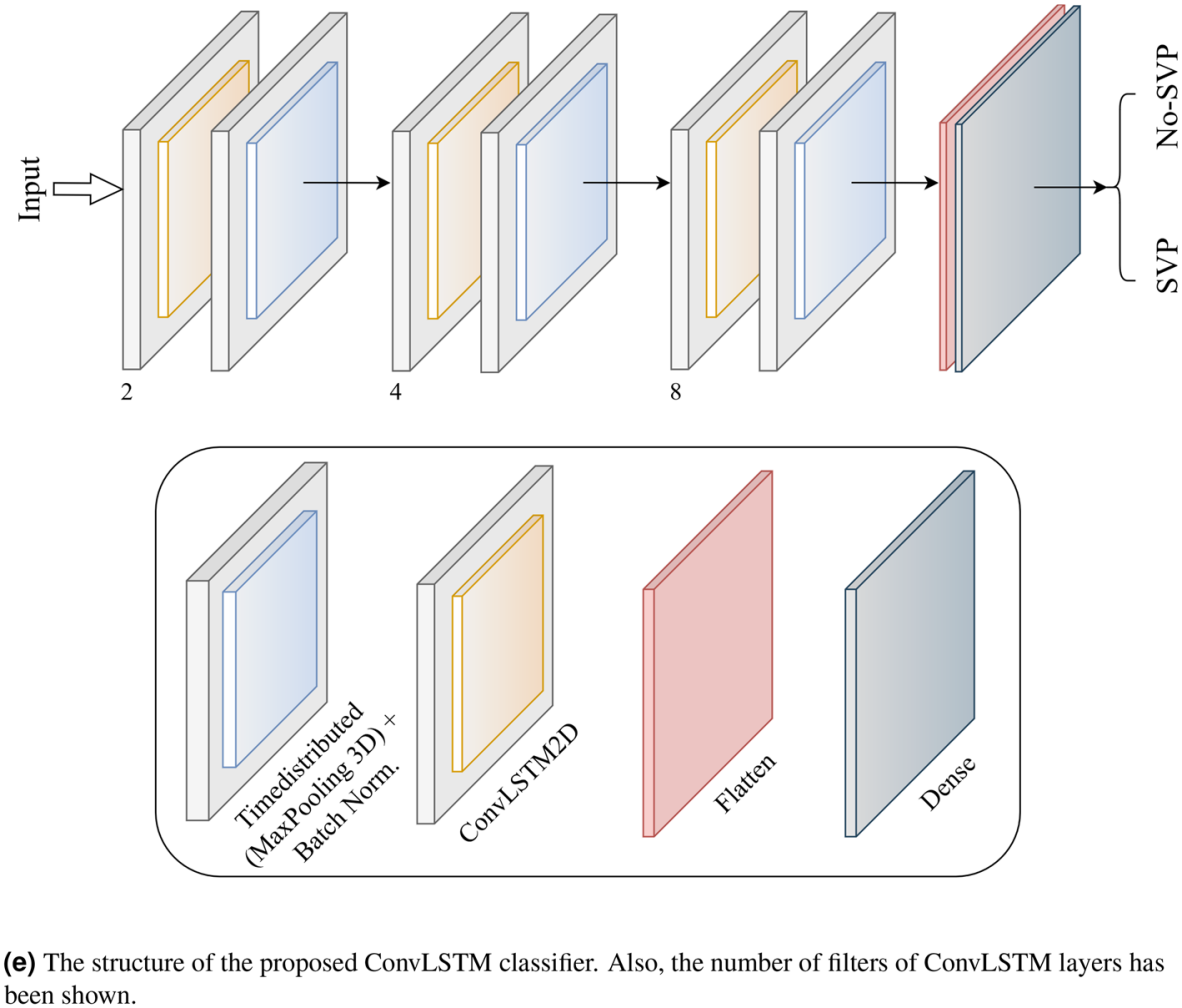
The classifier models that are used to detect SVP.

One of the classifier models used includes Inception modules^31^, 3D pooling, and 3D convolution layers to extract spatial-temporal features from the input videos in real-time. As shown in Fig. 6a, the 3D Inception-Based classifier block consists of different layers, including the input. Each Inception module is a combination of 3D convolution, batch normalization, and ReLU activation functions in which their outputs merge into a single vector and create the input of the next layer. Max-pooling layers support alternating convolutional layers. Also, the dropout layer is applied as a regularization operation to limit overfitting. Finally, fully connected layers are linked to an output layer which classifies the SVP status.

#### 3D Dense-ResNet

In this paper, we use the iterative advancement properties of ResNets to make densely connected residual networks for classifying SVP, which we call 3D Dense-Resnet. In FC DenseNets^32^, the convolution layers are densely connected, but in Dense-Resnet, we apply dense connectivity to ResNets modules. Therefore, the 3D Dense-Resnet model executes iterative advancement at each representation step (in a single ResNet) and utilizes dense connectivity to get refined multi-scale feature representations. Hence, by combining FC-DenseNets and FC-ResNets into a single model that merges the advantages of both architectures. This brings the architecture to use the advantages of both dense connectivity and residual patterns, namely: iterative refinement of representations gradient flow, multi-scale feature combination, and deep supervision^33^. The connectivity pattern of 3D Dense-ResNet is shown in Fig. 6b. First, the input is processed with a Conv3D convolution followed by a Max-Pooling 3D operation. After that, the output is fed to a Dense block organized by residual blocks based on ResNets.

The number of kernels in a convolution process in CNN is equal to that of the input maps used for the input. Also, to provide an output feature map of the layers, the outcomes add with a bias term; the procedure is repeated with various kernels to get the desired number of output feature maps. These convolution layers are followed by Batch Normalization and a Rectified Linear Unit (ReLU) and set to decrease the number of input feature maps at the output. Global-Average-Pooling and Dense layers followed the final output.

#### 3D ResNet

The proposed 3D ResNet network is based on the ResNets structure^34^. ResNets present shortcut connections that skip a signal from one layer to another layer. The connections transfer the gradient flows of the model from later layers to earlier layers, leading to facilitating the training process of deep models. The structure of the proposed 3D Resnet is shown in Fig. 6c.

First, the input is processed with a Conv3D convolution followed by Max-Pooling, Batch-Normalization, and a Rectified Linear Unit (ReLU) to decrease the number of input feature maps at the output. After that, the output is fed to a residual block organized by a skip connection, and to provide an output feature map of each layer, the outcomes add with a bias term. The number of kernels of Conv3D layers in residual blocks and in the first layer is $3 \times 3 \times 3$. All the other Conv3D layers in the 3D ResNet have kernel size of $1 \times 1 \times 1$. Finally, Global-Average-Pooling and Dense layers followed the final output.

#### Long-term recurrent convolutional network (LRCN)

Another method that can be utilized for the detection of SVP is a CNN model and LSTM model trained individually. To extract spatial features from the frames of the video, the CNN network can be used, and for this goal, a pre-trained model can be employed that can be fine-tuned for the issue. Then, the LSTM network can use features extracted from the previous model to predict the absence or presence of SVP in the video. But here, another method known as the Long-term Recurrent Convolutional Network (LRCN) has been used^35^, which integrates CNN and LSTM layers in a single network (Fig. 6d). The Convolutional layers are utilized for spatial feature extraction from the video frames, and after that, the spatial features are fed to the LSTM layer(s) at each time-steps. This process is Temporal sequence modeling, and the model directly learns spatiotemporal features in a robust end-to-end model.

Also, the TimeDistributed wrapper layer has been utilized, which provides usage of the same layer for every frame of the video separately. So it creates a layer that has the potential to take input of shape (*Num*-*of*-*Frames*, *Width, Height, Num*-*of*-*Channels*) if the layer’s input shape was (*Width*, *Height*, *Num*-*of*-*Channels*), which is very advantageous as it authorizes the input of the whole video into the network in a single shot. For training the proposed LRCN model, time-distributed Conv2D layers have been used, followed by Dropout layers and MaxPooling2D layers. Conv2D layers extract features and then will be flattened by using the Flatten layer. After that, the output will be fed to an LSTM layer. The Dense layer with activation of softmax will then apply the final result from the LSTM layer. In this model, the size of kernel size of Conv2D layers is 3 $\times$ 3, and the pooling size of MaxPooling2D is $2 \times 2$.

#### ConvLSTM

The other approach proposed for detecting the presence or absence of SVP is a combination of ConvLSTM cells. A ConveLSTM cell is a kind of an LSTM model that includes convolutions functions in the model. It is an LSTM with convolution infixed in the network, which makes it apt to identify spatial features of the data while considering the temporal relation. This method effectively catches the spatial connection in the individual frames and the temporal connection across the various frames for video classification. Consequently, the ConvLSTM can take in 3D (*Width*, *Height*, *Num*-*of*-*Channels*) as input in this convolution network, whereas a simple LSTM takes in 1D input.

**Figure 7:**
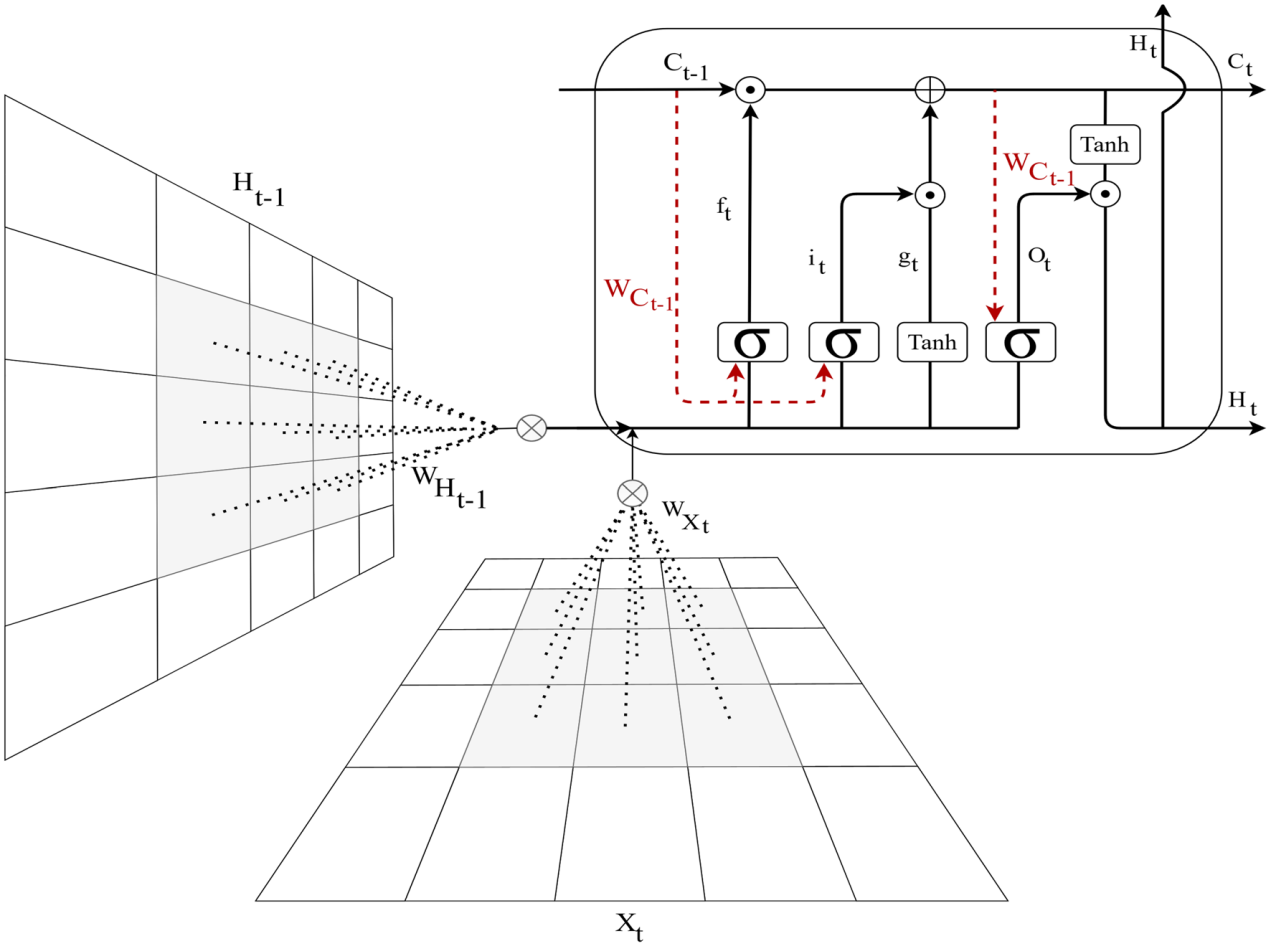
Structure of the convolutional LSTM (ConvLSTM) cell.

The overall structure of the proposed ConvLSTM cell is shown in Fig. 7, where $\sigma$ is the sigmoid function, *W* is presented as the weight for each layer, *b* is the bias, $X_t$ is the input in time step *t*, and the hyperbolic tangent function is represented by the *tanh*. Also, the Hadamard product operator is shown by $\bigodot$, $f_t$ is forget gate, $c_t$ is the cell state, $i_t$ is the input gate, and $O_t$ is the output gate.

The value obtained by taking the sigmoid function after getting $x_t$ and $h_{t-1}$ is equal to the value that the forget gate sends out. The range of the sigmoid function output is from 0 to 1. Information from the previous cell is forgotten if the output value is 0, and if it is 1, information from the previous cell is wholly memorized. Also, $i_t \bigodot g_t$ is a gate for holding current information and catches $h{t-1}$ and $x_t$, and uses the sigmoid function.

After that, the value that takes the Hadamard product operation and Hyperbolic Tangent (tanh) function is sent from the input gate. As the range of $g_t$ is from -1 to 1 and $i_t$ is from 0 to 1, each represents the direction and intensity of storing current information. The formula of *ConvLSTM* cell is shown in what follows:

4 $$\begin{aligned} f_t= & {} \sigma (W_{Xf} *X_t +W_{Hf} *H_{t-1} + W_{cf} \odot C_{t-1} + b_f) \end{aligned}$$

5 $$\begin{aligned} i_t= & {} \sigma (W_{Xi} *X_t +W_{Hi} *H_{t-1} + W_{ci} \odot C_{t-1} + b_{Hi}) \end{aligned}$$

6 $$\begin{aligned} g_t= & {} \tanh (W_{Xg} *X_t +W_{Hg} *H_{t-1} + b_{h-g}) \end{aligned}$$

7 $$\begin{aligned} C_t= & {} (f_t \odot C_{t-1}) + (i_t \odot g_{t}) \end{aligned}$$

8 $$\begin{aligned} O_t= & {} \sigma (W_{Xo} *X_t +W_{Ho} *H_{t-1} + W_{Co} \odot C_{t} + b_{h-o}) \end{aligned}$$

9 $$\begin{aligned} H_t= & {} o_t \odot \tanh (c_{t}) \end{aligned}$$

The cell state *H*, input gate *i*, output gate *O*, cell output *C*, cell input *X*, and forget gate *f* are all 3D tensors while in the original LSTM, where all these elements were 1D vectors. Also, all matrix multiplications are considered by operations’ convolution, which shows that the number of presented weights in all *W* in each cell can be less than in the original LSTM^36^.

In our proposed model, *ConvLSTM*2*D* has used Keras layers. Also, the *ConvLSTM*2*D* layer catches the number of kernels and filters size needed for using the convolutional processes. The outcome of the layers, in the end, is flattened and after that is fed to the Dense layer with *SoftMax* activation. Also, *MaxPooling*3*D* layers have been used to decrease the sizes of the frames and avoid unneeded calculations and Dropout layers to control the overfitting of the proposed model.

As the architecture is simple, the number of trainable parameters is small. The overall structure of our proposed method based on ConvLSTM is shown in Fig. 6e. The kernel size of *ConvLSTM*2*D* is $3 \times 3$, and the Hyperbolic Tangent (Tanh) activation function is applied for ConvLSTM2D layers. After each ConvLSTM2D layer, MaxPooling3D layers with pooling sizes of $1 \times 2 \times 2$ and Batch Normalization layers have been applied. The final result has passed from Flatten and Dense layers.

To analyze the best performance of every classifier model, we ran several different experiments modifying the number of epochs, batch size, and learning rate. Table 1 summarizes the characteristics of the proposed classifiers.

**Table 1:** Characteristics of the U3D-Net classifiers.

	3D inception	3D Dense-ResNet	3D ResNet	LRCN	ConvLSTM
Batch size	30	30	30	30	30
Number of epochs	100	100	100	100	100
Learning rate	0.0003	0.0028	0.002	0.0029	0.004
Optimizer	Adam	Adam	Adam	Adam	Adam

## Results

For interpreting the performance of every model, various metrics have been utilized, which include some parameters. These parameters are TP, TN, FP, and FN, which refer to the true positive, true negative, false positive, and false negative, respectively. Accuracy, Precision, Recall, Specificity, F1-score, Negative Predictive Value (NPV), Dice score, and Intersection-Over-Union (IOU) have been used for evaluating the proposed models^37–39^.

### U3D-Net localizer result

To evaluate the proposed optic disc localizer model, the Dice score and IOU have been used. Dice Score was applied as a statistical validation factor to measure the similarity between the manual segmentation map and the final segmentation map of the model. IOU is a factor utilized to define the area of overlap between two regions. The greater the region of overlapping brings greater the IOU factor. IOU and Dice factors utilize different methods to calculate how matching an image segmentation algorithm’s outcomes are to its ground truth segmentation map.

**Figure 8:**
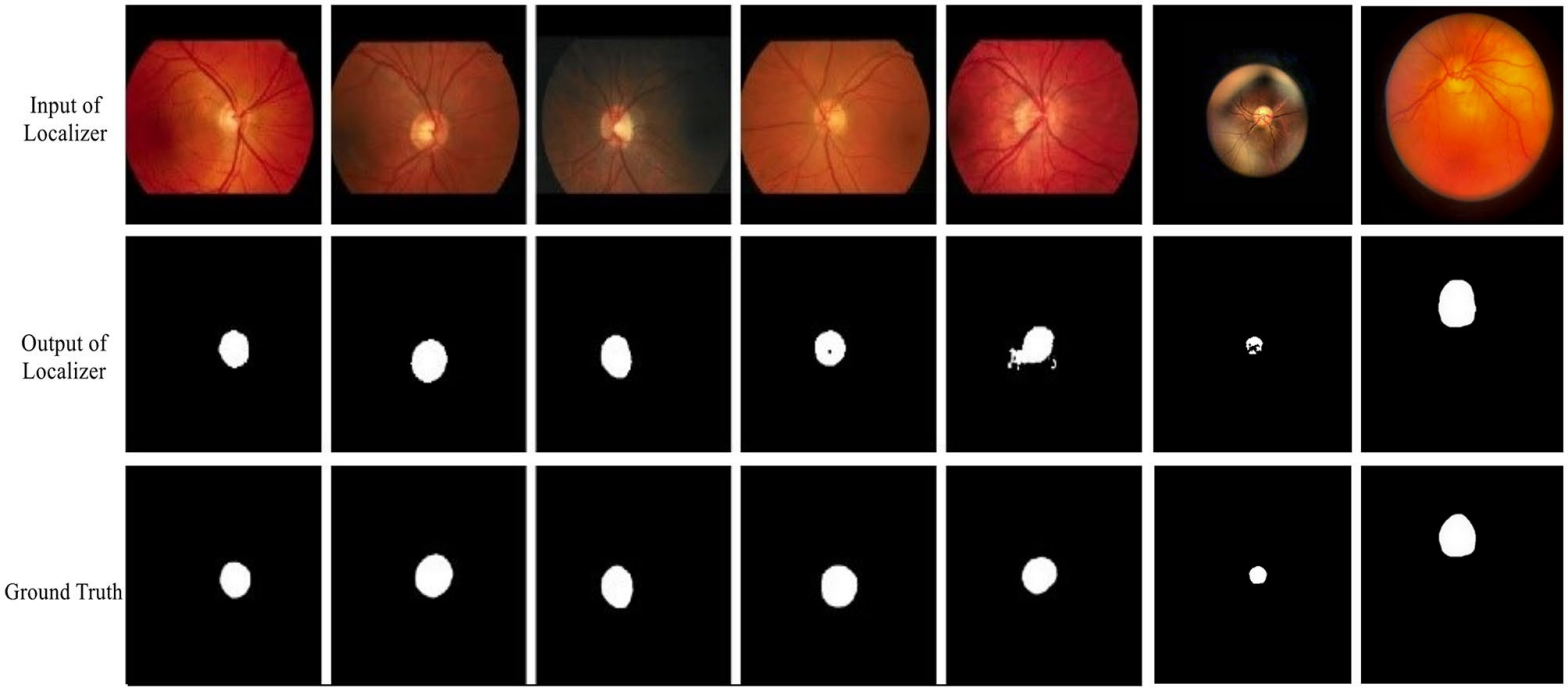
Segmentation maps created by the optic disc localizer versus the ground truth ones.

Figure 8 is a sample comparison of the optic disc segmentation maps extracted by the model with the ground truth. The localizer model was able to achieve a Dice score of 0.95 and an IOU of 0.91 (Table 2). Also, with regard to the (partial) evaluation of localization performance on the actual dataset, we randomly selected a number of samples from the dataset. The average Dice and IOU scores for the given samples were 0.87 and 0.84, respectively.

**Table 2:** Comparison of the proposed model with existing models for optic disc localization using the DRIONS-DB dataset.

Methods	Dice	IOU	Prediction time(s)	Hardware settings
Mannis et al.^40^	0.97	0.94	0.65	NVIDIA TITAN-X GPU
Ramani et al.^41^	0.82	0.89	1.41	Intel(R) Core(TM), 1.70 GHz
Morales et al.^42^	0.90	0.84	–	–
Sevatoplsky et al.^43^	0.94	0.89	0.13	NVIDIA GRID (Kepler GK104)
Walter et al.^44^	0.68	0.62	–	–
U-Net	0.94	0.88	0.11	NVIDIA Tesla K80
Attention U-Net	0.94	0.89	0.19	NVIDIA Tesla K80
U3D-Net Localizer	0.95	0.91	0.03	NVIDIA Tesla K80

### U3D-Net classifiers result

Table 3 summarizes the Sensitivity, Specificity, Precision, Accuracy, F-1 Score, and Negative Predictive Value attained for each classifier model utilizing the abovementioned parameters. For evaluating the classifier models, K-fold cross-validation has been used. It typically results in a less biased model compared to other techniques, as every observation from the dataset has the opportunity to come into view in training and test set^45^. Our results demonstrate that the 3D Inception achieved better results.

**Table 3:** General performances and comparison of proposed classifier models with the existing research.

		Sensitivity (%)	Specificity (%)	Precision (%)	Accuracy (%)	F1 Score (%)	NPV (%)	Mean ROC (%)
3D Inception	Fold1	78	93	93	85	85	77	96
	Fold2	99	87	76	91	86	99	98
	Fold3	91	77	68	82	78	94	95
	Fold4	88	88	88	88	88	88	96
	Fold5	99	66	55	76	71	99	99
	**Ave.**	**90** ± **8**	**82** ± **9**	**76** ± **13**	**84** ± **5**	**81** ± **6**	**91** ± **8**	**96.2**
3D Dense-ResNet	Fold1	64	50	76	60	69	35	53
	Fold2	58	70	82	61	68	41	69
	Fold3	62	61	58	61	60	64	71
	Fold4	88	87	88	88	88	87	95
	Fold5	99	68	52	76	69	99	98
	**Ave.**	**74** ± **16**	**67** ± **12**	**71** ± **13**	**69** ± **11**	**70** ± **9**	**65** ± **24**	**77.2**
3D ResNet	Fold1	57	50	95	57	71	6	76
	Fold2	36	66	75	44	48	27	56
	Fold3	65	40	86	61	74	16	52
	Fold4	67	50	86	64	76	25	62
	Fold5	48	99	99	50	65	5	64
	**Ave.**	**54** ± **11**	**61** ± **20**	**88** ± **8**	**55** ± **7**	**66** ± **10**	**15** ± **9**	**62**
LRCN	Fold1	73	85	78	80	75	80	88
	Fold2	69	81	88	73	78	56	93
	Fold3	91	99	99	94	95	84	99
	Fold4	95	99	99	97	97	93	99
	Fold5	73	87	95	76	82	50	84
	**Ave.**	**80** ± **10**	**90** ± **7**	**91** ± **7**	**83** ± **9**	**85** ± **8**	**72** ± **16**	**92.6**
ConvLSTM	Fold1	72	76	76	74	74	72	84
	Fold2	69	99	99	79	82	61	87
	Fold3	69	90	94	76	80	58	92
	Fold4	88	75	80	82	84	85	88
	Fold5	95	99	99	97	97	91	99
	**Ave.**	**78** ± **10**	**87** ± **10**	**89** ± **9**	**81** ± **8**	**83** $\pm$ **7**	**73** ± **12**	**90**
Laurent et al. (Two observers)^46^	**Observer1**	84	89	90	86	–	82	–
	**Observer2**	76	68	75	73	–	70	–

**Figure 9:**
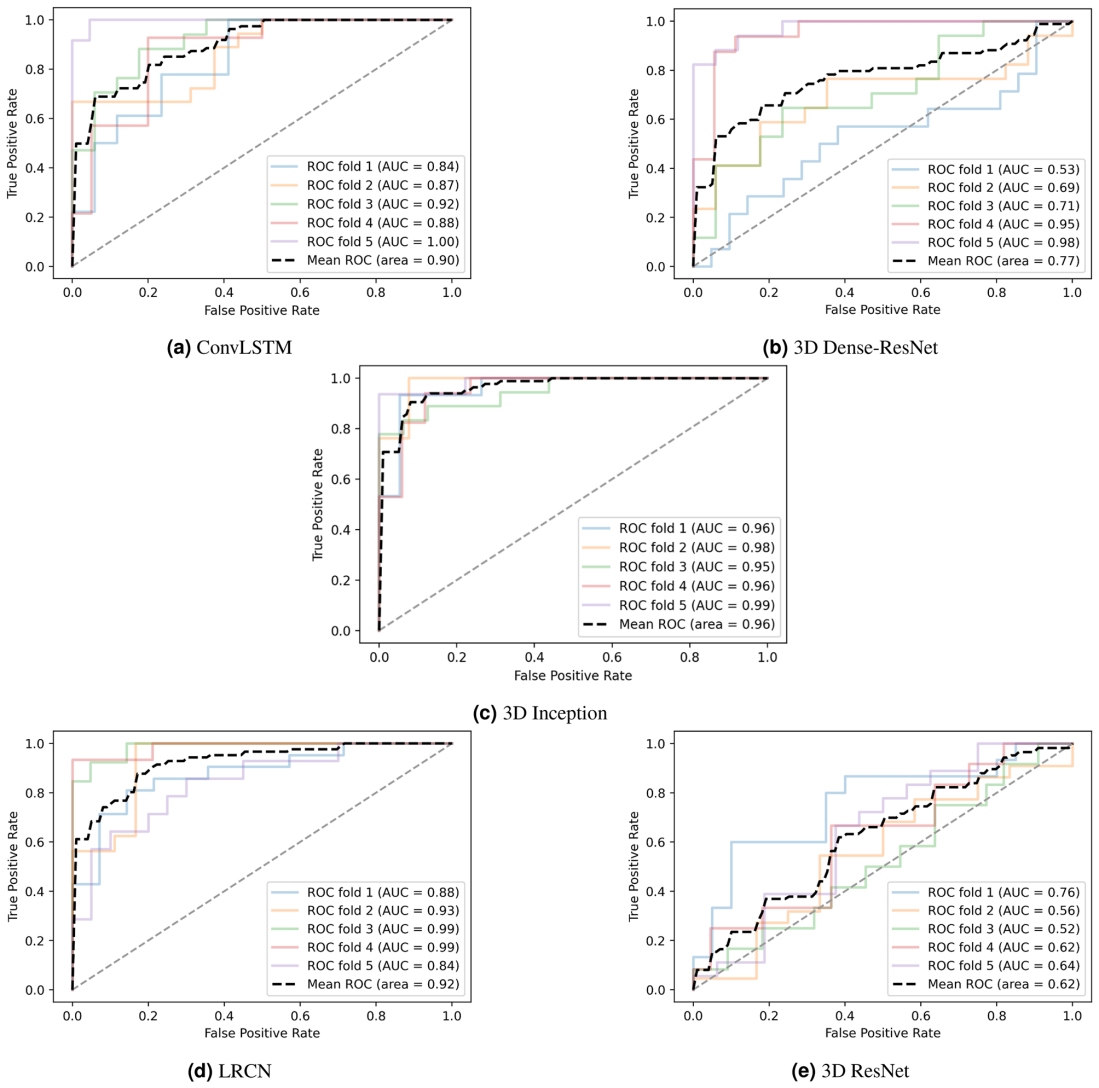
The receiver operating characteristic (ROC) curves for all the different classifiers.

In Fig. 9 we compared the area under the Receiver Operating Characteristic (ROC) curves to assess each model’s performance in separating the presence and absence of SVP.

## Discussion

In this study, we have developed a model based on a recurrent residual U-Net that utilizes an attention mechanism to autonomously and objectively classify SVPs as present or absent in fundus videos. To the best of our knowledge, this is the first study to use a deep neural network for SVP assessment, and as a result, our findings set the first benchmark for SVP assessment using such an approach. While there have been previous attempts to use computer-aided analysis for quantifying SVP amplitudes^23,47,48^, they are resource-intensive and require post-video capture analysis. Our solution overcomes these shortfalls by providing an approach called U3D-Net that can readily analyze fundus videos and provide a binary classification of SVP status (i.e., present or absent). As SVPs are particularly visible in the optic disc area, the ablation of the U3D-Net model (i.e., optic disc localizer) significantly decreased the performance of the overall model. This is due to the fact that the network considered the entire region of the fundus images without any specific focus on the optic disc, where physiological SVPs are known to occur. We proposed a deep learning model based on U-Net to detect the optic discs, and the images of the segmented optic discs were then fed to another time-series deep learning model to classify SVPs on fundus videos. In order to select the best model with the highest performance for the classification task, we trained different time series models including the 3D Inception, 3D Dense-ResNet, 3D ResNet, LRCN, and ConvLSTM. By comparing these models’ performances (Table 3), the 3D Inception model outperformed the others, achieving a sensitivity of 90 ± 8% in classifying SVPs.

This is comparable to a recent clinical study^46^ that reported a sensitivity of 84.7% and 76.8% for two expert clinicians that had subjectively classified SVPs as present or absent. Our model achieved a specificity of 82 ± 9%, and, once again, this is comparable to the aforementioned study in which the same expert clinicians scored 89.2% and 68.6% for specificity, respectively. Finally, the accuracy of our model was 84 ± 5% in comparison to 73.1% and 86.7% accuracy achieved by expert clinicians in the same study. Collectively, despite the relatively low sample size used to train and evaluate our model, we have demonstrated that it is possible to develop a deep learning framework that can achieve a sensitivity, specificity, and accuracy comparable to that of expert clinicians with further room for improvement if a larger sample size is used.

SVP analysis can provide significant clinical insight into the hemodynamic status of the optic nerve head. Due to its anatomical location, SVPs observed in the central retinal vein are a direct result of the hemodynamic interaction between the intraocular and intracranial pressure^49^. Accordingly, SVP analysis can reveal blood flow dysfunction due to ocular or neurological conditions^50^. Traditionally, SVPs have been assessed by an ophthalmologist in the clinic using a 78D or 90D ophthalmic lens. However, the major limitation of such an approach is that it subjectively assesses SVP, and subtle vein pulsations can easily be missed. This is evident through previous studies that have reported a varying degree of SVP presence in normal and glaucoma patients^8^. However, studies that have used computer-aided analysis of retinal videos have demonstrated that SVPs are identifiable in almost 100% of the population and that it’s the pulse amplitudes that can vary between nonexistent (i.e., < 15% diameter expansion) to clinical evidence (i.e., > 50% diameter expansion)^23,46^. Our study has a few limitations. First, we have used a relatively small sample size to train and evaluate our deep learning model. Despite this, our model performance is comparable to that of expert clinician grading. Second, we have used subjective grading as our ground truth. While an exact SVP amplitude produced by a computer-aided image analysis program could have been used in our study, we purposely decided to use subjective grading as our ground truth mainly for two reasons:

- Subjective assessment is an established method in the clinic and thus our findings can directly be translated into a clinical setting.
- Compare our findings to an available subjective study.

Finally, we have used a binary classification for SVP analysis. While a multi-tier grading of SVPs can inform enhanced clinical decision-making, binary classification of SVPs can lay the foundation for future work in this area, all whilst providing evidence on the overall hemodynamic status of the optic nerve head.

## Conclusion

In conclusion, we have developed a deep learning model, named U3D-Net, to objectively analyze retinal fundus videos and readily provide an autonomous classification for SVP presence or absence. Our highest performance model achieved a sensitivity, specificity, and accuracy of 90 ± 8%, 82 ± 9%, and 84 ± 5% in classifying SVPs. This serves as an initial benchmark for similar studies that may be carried out in the future. With a significant increase in imaging technologies, our model can be integrated into portable fundus ophthalmoscopes and be used to scan for SVP presence. However, further studies with a larger and heterogeneous sample size as well as multi-class labeling are needed to fully exploit the clinical benefits of autonomous SVP classification.

## Data Availability

The retinal video dataset generated and/or analysed during the current study is not publicly available due to limitations imposed on the study’s ethics approval but is available from the corresponding author on reasonable request and following an ethics approval process.
